# Comprehensive Review on Chiral Stationary Phases in Single-Column Simultaneous Chiral–Achiral HPLC Separation Methods

**DOI:** 10.3390/molecules29061346

**Published:** 2024-03-18

**Authors:** Lajos Attila Papp, Zoltán István Szabó, Gabriel Hancu, Lénárd Farczádi, Eleonora Mircia

**Affiliations:** 1 Department of Pharmaceutical Chemistry, Faculty of Pharmacy, “George Emil Palade” University of Medicine, Pharmacy, Science and Technology of Târgu Mures, 540142 Târgu Mureș, Romania; lajos.papp@umfst.ro; 2 Department of Drugs Industry and Pharmaceutical Management, Faculty of Pharmacy, “George Emil Palade” University of Medicine, Pharmacy, Science and Technology of Târgu Mures, 540142 Târgu Mureș, Romania; zoltan.szabo@umfst.ro (Z.I.S.); eleonora.mircia@umfst.ro (E.M.); 3 Sz-Imfidum, Ltd., Sat Lunga nr. 504, 525401 Târgu Mures, Romania; 4 Chromatography and Mass Spectrometry Laboratory, Centre for Advanced Medical and Pharmaceutical Research, “George Emil Palade” University of Medicine, Pharmacy, Science and Technology of Târgu Mures, 38 Gheorghe Marinescu Street, 540142 Targu Mures, Romania; lenard.farczadi@umfst.ro

**Keywords:** high-performance liquid chromatography, simultaneous separation, enantioselective, chemoselective, chiral stationary phase, separation mechanism

## Abstract

This comprehensive review explores the utilization of chiral stationary phases (CSPs) in the context of single-column simultaneous chiral–achiral high-performance liquid chromatography (HPLC) separation methods. While CSPs have traditionally been pivotal for enantioselective drug analysis, contemporary CSPs often exhibit notable chemoselective properties. Consequently, there is a discernible trend towards the development of methodologies that enable simultaneous enantio- and chemoselective separations utilizing a single CSP-based chromatographic column. This review provides an exhaustive overview of reported HPLC methods in this domain, with a focus on four major CSP types: cyclodextrin-, glycopeptide antibiotic-, protein-, and polysaccharide-based CSPs. This article delves into the diverse applications of CSPs, encompassing various chromatographic modes such as normal phase (NP), reverse phase (RP), and polar organic (PO). This review critically discusses method development, emphasizing the additional chemoselective separation mechanisms of CSPs. It also explores possibilities for method optimization and development, concluding with future perspectives on this evolving field. Despite the inherent challenges in understanding the retention mechanisms involved in chemoselective separations, this review highlights promising trends and anticipates a growing number of simultaneous enantio- and chemoselective methods in pharmaceutical analyses, pharmacokinetic studies, and environmental sample determinations.

## 1. Introduction

Chirality plays a pivotal role in the pharmacological activity of many compounds, as the distinct spatial arrangement of enantiomers can significantly influence their biological effects. In the context of modern drug development and analysis, the enantioseparation of chiral compounds is an essential aspect. In many cases, the pharmacological impact of a chiral drug is attributed to one enantiomer (eutomer), while the other enantiomer (distomer) usually exhibits lower activity, but sometimes can generate adverse or toxic effects. Furthermore, the trend of introducing enantiomerically pure drugs into therapy has been on the rise in the last two decades [1,2,3]. Regulatory agencies, such as the Food and Drug Administration (FDA) and European Medical Agency (EMA), published specific guidance on the topic in the early 1990s, requiring the evaluation of pharmacological profiles of chiral drugs for both the racemate and individual enantiomers [4,5].

High-performance liquid chromatography (HPLC) is the golden standard in the analysis of drugs from different biological, pharmaceutical, or environmental samples in both chiral and achiral investigations [6,7]. In the general approach, separate analytical methods are applied for the chiral and achiral analysis of the same matrix; however, in many cases, the quantification of different chiral structurally related compounds is necessary. The determination of the parent drug and its metabolites in pharmacokinetic studies, the purity control of drugs in pharmaceutical analysis, or the determination of chiral pharmaceuticals from environmental samples are suggestive examples.

To resolve this problem and to avoid interferences from different analytes when using an enantioselective method, several approaches can be adopted. Two-dimensional liquid chromatography, including comprehensive (all sample components are transferred from the first to the second dimension) and heart-cut (only a fraction eluted from the first dimension is transferred to the second one) approaches, as well as in-line column coupling methods are commonly applied techniques [8,9]. However, the easiest solution would be the application of a single column with appropriate selectivity for both types of separation. The use of a chiral stationary phase (CSP) that provides good chemoselectivity remains the best choice in terms of the analysis time, solvent consumption, and the simplicity of analytical infrastructure.

The application of CSPs in simultaneous chemo- and enantioselective tests is gaining popularity, which can be due to the increased stability, efficiency, and higher chemoselectivity of the novel CSPs. In addition, a high variety of CSPs are already available, most of them showing compatibility with different eluent types. These facts facilitate the analysis of compounds belonging to structurally different drug classes [10].

This review offers a comprehensive overview of methods available for the simultaneous chiral and achiral separation of drug compounds, their related substances, and metabolites, utilizing a single chromatographic column. The study encompasses four crucial types of CSPs: cyclodextrin (CD)-, glycopeptide antibiotic-, protein-, and polysaccharide-based columns, with a specific focus on their additional chemoselective capabilities. Following a critical presentation of the existing methods, the review delves into general aspects of the studied CSP types and explores potential retention mechanisms implicated in chemoselective separations.

This review not only serves to consolidate the current state of the art, but also endeavours to outline emerging trends and future perspectives in this dynamic and rapidly evolving area of HPLC separations. Through this exploration, we aim to contribute valuable insights to researchers engaged in the pursuit of efficient and selective chiral–achiral HPLC separation methodologies.

## 2. Enantio- and Chemoselective Methods

The simultaneous separation of chiral and achiral compounds on a single chromatographic column has gained considerable attention due to its efficiency and practicality. This approach becomes particularly crucial in pharmaceutical analysis, where the enantiomeric purity of drugs can profoundly impact their efficacy and safety. In recent years, advancements in chromatographic techniques and the development of diverse CSPs have opened new possibilities for achieving simultaneous chiral–achiral separations with enhanced selectivity and efficiency.

A normal phase (NP) HPLC method was reported in 1992 by Fieger and Blaschke for the chiral separation of the calcium channel blocker verapamil and its main metabolite norverapamil, in human plasma samples, after oral administration, using amylose(tris-3,5-dimethylphenylcarbamate (ChiralPak AD) as a CSP and *n*-hexane:isopropranol (90:10, *v*/*v*) as a mobile phase. Another method using an α1-acid glycoprotein (Chiral-AGP) CSP in combination with a mobile phase consisting of phosphate buffer (pH 7.0):acetonitrile (90:10, *v*/*v*) was also developed for the same purpose; however, this method required a preanalytical step involving the off-line acetylation of norverapamil, to avoid the overlapping of its peaks with those of verapamil. As a structural analogue of verapamil, gallopamil was separated on both columns under similar analytical conditions [11].

An improved method using a Chiral-AGP column with a phosphate buffer (pH 6.5):acetonitrile (91:9, *v*/*v*) eluent for the simultaneous determination of verapamil and norverapamil enantiomers from human plasma was developed by Stagni and Gillespie. Before the analysis, norverapamil was acetylated to N-acetylnorverapamil during the extraction procedure. The limits of quantitation (LOQ) were 3 ng/mL for verapamil and 2 ng/mL for norverapamil. The validated method proved to be applicable for the study of the stereoselective disposition of the verapamil after oral administration [12].

The chiral separation capability of the glycopeptide antibiotic teicoplanin-based CSP on 54 different amino acids as well as on several dipeptides was evaluated by Berthod et al. Adequate selectivity between amino acid enantiomers could be obtained in most cases by applying hydro-organic mobile phases without the addition of any acidic or basic modifier. Moreover, the stereoisomers of some dipeptides with two chiral centers could be resolved using the same approach. Based on the experimental results and molecular modeling data, the authors revealed that a primary electrostatic interaction occurs between the protonated amino group of teicoplanin and the carboxyl anion of the amino acid/dipeptide, while secondary interactions such as hydrogen-bonding, hydrophobic, and dipole-dipole interactions provide enantiomeric selectivity to the complexes. Moreover, the distance between the anionic group of the analyte, involved in the primary interaction with the chiral selector (CS), and the chiral center may have a crucial effect on the chiral resolution [13].

The chiral and achiral separation of verapamil and seven of its metabolites was studied by Brandšteterová and Wainer using an α1-AGP column and eluents containing different proportions of phosphate buffer and acetonitrile. To enhance sample clean-up and preconcentration, both off-line solid-phase extraction (SPE) using SepPak C18 cartridges and on-line SPE with a semipermeable surface SDS C8 pre-column was implemented. LOQ values were in the range of 1–5 ng/mL for all compounds. By applying the method, the stereoselectivity of pharmacokinetics for verapamil and norverapamil was confirmed [14].

A cellobiohydrolase-based CSP was employed by Zhang et al. for the simultaneous determination of the β2-adrenoreceptor agonist salmeterol and its metabolite α-hydroxy-salmeterol following incubation in human liver microsomes of the racemic and enantiomeric pure *R*- and *S*-salmeterol, respectively. The effect of the pH, buffer concentration, and isopropanol proportion in the mobile phase on the enantioselectivity and chromatographic retention were investigated. The increase in the buffer or isopropanol concentration caused an increase in the enantioselectivity and a decrease in the retention time while elevating the pH of the buffer which increased both analytical responses. It was demonstrated that under the applied experimental conditions, α-hydroxy-salmeterol is the only metabolite produced and the oxidative transformation of the parent drug is not stereoselective. The enantiomeric elution order of salmeterol and its hydroxylated derivative was found to be identical, with the *R*-enantiomer migrating first, followed by the *S*-enantiomer [15].

An NP HPLC bioanalytical method using a cellulose-based CSP capable of the chiral separation of the general anesthetic drug ketamine and its main metabolite norketamine in human plasma was developed by Yanagihara et al. Plasma samples underwent a liquid–liquid extraction (LLE) procedure to avoid the appearance of interfering peaks from the plasma matrix. Stereoselective separation was accomplished using a Chiralcel OD column with a mobile phase composed of *n*-hexane:isopropanol (98:2, *v*/*v*). The method exhibited limits of detection (LOD) of 5 ng/mL for ketamine and 10 ng/mL for norketamine. This method proved to be applicable to the stereoselective monitoring of the pharmacokinetics of ketamine after its oral administration [16].

A bioanalytical method quantification of 10-hydroxycarbazepine and carbamazepine-10,11-*trans*-dihydrodiol enantiomers, the two major metabolites of oxcarbamazepine, was developed by Volosov et al. Enantiomeric resolution was achieved on a Diacel Chiralcel OD column under isocratic conditions, using *n*-hexane:ethanol:isopropanol (18:2:1, *v*/*v*/*v*) as the mobile phase, with the addition of glacial acetic acid (0.1%). This method provides a reliable means for determining the enantiomers of 10-hydroxycarbazepine and carbamazepine-10,11-*trans*-dihydrodiol in human urine, with good recoveries within 20 min, with an LOQ of 0.2 mg/L and 0.4 mg/L, respectively [17].

Kaddoumi et al. developed two HPLC methods with fluorescence detection for the simultaneous determination of sympathomimetic amines in spiked human plasma. The first method involved ephedrine, norephedrine, 2-phenylethylamine, 4-bromo-2,5-dimethoxyphenylethylamine, phentermine, and fenfluramine. The second method was an enantioselective one, specifically designed for the separation of dexfenfluramine, levofenfluramine, and their active metabolites dexnorfenfluramine and levonorfenfluramine. In the extraction process, analytes were extracted from plasma at pH 10.6 using ethyl acetate, with fluoxetine serving as the internal standard (I.S). The extracts underwent evaporation and were then derivatized with the fluorescence reagent 4-(4,5-diphenyl-1H-imidazole-2-yl)benzoyl chloride in the presence of carbonate buffer (pH 9.0). Gradient separation was achieved using a C18 column for the achiral separation and a Chiralcel OD-R column for the chiral separation. The chiral method was successfully applied to determine the enantiomers of fenfluramine and norfenfluramine, alongside phentermine, in rat plasma following the intraperitoneal administration of racemic fenfluramine and phentermine concomitantly [18].

The simultaneous chiral analysis of 15 different proteinogenic and non-proteinogenic amino acids was reported using a teicoplanin CSP and isocratic elution (acetonitrile:water, 75:25, *v*/*v*) coupled to triple quadrupole MS using an ion spray ionization technique, by the post-column addition of formic acid to the eluent, and a multiple reaction monitoring (MRM) detection mode. Tandem MS-MS, owing to its specificity, allows for the simultaneous determination of co-eluting enantiomers of various amino acids. Therefore, the authors paid special attention to the separation of amino acid isomers having the same parent and product ions, such as *L*,*D*-Leu/Ile or *L*,*D*-Val/Iva. The effect of the organic modifier (acetonitrile, methanol) proportion on the separation was examined, and an increasing retention time was observed when the concentration of the organic component in the eluent was increased. The LOD ranged from 2.5 to 50 μg/L, depending on the specific amino acid being analyzed. According to these results, teicoplanin-based CSP provides not only good enantioselectivity, but also good chemoselectivity in terms of the separation of positional isomers and structurally different amino acids [19].

A chiral HPLC method has been developed to analyze the enantiomers of N-ethyl-3,4-methylenedioxyamphetamine and its metabolites, including *O*-glucuronyl-N-ethyl-4-hydroxy-3-methoxyamphetamine and 3,4-methylenedioxyamphetamine in human plasma. The chiral discrimination of these compounds was done by employing β-CD as a mobile phase additive for N-ethyl-3,4-methylenedioxyamphetamine and 3,4-methylenedioxyamphetamine, and a protein-based chiral column (Chiral-CBH) for *O*-glucuronyl-N-ethyl-4-hydroxy-3-methoxyamphetamine. The LOQ for the studied enantiomers and their metabolites in plasma were between 1.2 and 16 ng/mL. The developed methods were applied to a pharmacokinetic study demonstrating the stereoselective metabolism of the new amphetamine analog. Significantly different plasma concentrations of the examined stereoisomers were observed. While the *R*-enantiomer of the parent substance predominated in the plasma samples investigated, higher concentrations of the *S*-enantiomers of the metabolites were measured [20].

An HPLC method for the simultaneous analysis of the proton pump inhibitor (PPI) lansoprazole and its primary metabolites, 5-hydroxy-lansoprazole and lansoprazole sulfone (achiral compound), was reported by Katsuki et al. using a cellulose-tris(3,5-dimethylphenyl)carbamate-based CSP (Chiralcel OD-R) under reversed phase (RP) conditions. The LOQ for both lansoprazole enantiomers were 0.25 μM and for their metabolites, 0.13 μM. Unfortunately, important details regarding the development of the method, such as the column and mobile phase selection, or the optimization of chromatographic conditions were not presented in the article. However, the validated method proved to provide the baseline separation for all compounds of interest and was applied in the study of the stereoselective metabolism of lansoprazole on human liver microsomes. The findings of the in vitro metabolic study were reported by the same research group in another paper, confirming the preferential stereoselective transformation of *S*-lansoprazole to the sulfone-derivative on the CYP3A4 isoenzyme [21].

A homemade cellulose-[tris-(3,5-dichlorophenylcarbamate)]-based chiral column was applied to the simultaneous enantiomeric separation of calcium channel blocking *cis*-diltiazem and its desacetyl metabolite. Interestingly, no simultaneous enantiomeric separation of both compounds was achieved in the NP or RP modes, and the purpose of the study could only be achieved under polar organic (PO) conditions. The analyte peaks were identified by an off-line electrospray ionization (ESI)-MS technique. The same study involved the development of an alternative capillary electrophoresis method (CE). The CE approach was tested for the quantitative detection of small enantiomeric impurities in pharmacologically used *cis*-diltiazem [22].

A cellobiohydrolase-based CSP was developed by Buechler et al. for the stereoselective determination of N-ethyl-3,4-methylenedioxyamphetamine and its two main metabolites from human plasma and urine samples after oral administration. The sample pretreatment involved the enzymatic hydrolysis of o-glucuronate and sulfate conjugates followed by SPE with a cation exchange phase. The extraction recoveries were above 92% in all cases, while the fluorimetric detection provided LOQ values of 5 ng/mL (N-ethyl-3,4-methylenedioxyamphetamine and 3,4-methylenedioxyamphetamine) and 10 ng/mL (N-ethyl-4-hydroxy-3-methoxyamphetamine). However, the method provided no baseline separation for all compounds, particularly N-ethyl-3,4-methylenedioxyamphetamine enantiomers showing only partial resolution, which could make it difficult to quantify both enantiomers in a single procedure. The results of the pharmacokinetic investigation performed in the same study showed a higher plasma half-life for the *R*-enantiomer, while the plasma concentration of the *S*-forms of the metabolites was significantly higher than those of their antipodes [23].

A cellulose-based CSP under RP conditions was employed by Gatti et al. for the simultaneous chiral analysis of a selective serotonin reuptake inhibitor (SSRI), fluoxetine, and its main active metabolite, norfluoxetine. The validated method proved to be suitable for therapeutical drug monitoring. The determination used LLE into acetonitrile:*n*-hexane:izopropanol, followed by re-extraction into phosphoric acid for cleanup. The analytes were separated using a Chiralcel OD-R column and a mobile phase containing potassium hexafluorophosphate and acetonitrile. During the evaluation of method specificity, the authors considered the possibility of the interference of several co-administered drugs, such as other antidepressants, anticonvulsants, and antipsychotics, and recommended different approaches in terms of sample pretreatment and minimal modifications in chromatographic conditions to avoid this problem [24].

The simultaneous chiral separation of lansoprazole, 5-hydroxy-lansoprazole, and lansoprazole-sulfone from human plasma has been performed by RP-HPLC by Miura et al., applying a phenylcarbamate β-CD-based CSP. The bioanalytical method was applied for a pharmacokinetic study, with *R*-lansoprazole showing a more advantageous pharmacokinetic profile than *S*-lansoprazole. Very similar separation conditions were successfully applied in a subsequent study for the simultaneous enantiomer quantitation of another PPI, rabeprazole, the enantiomers of its desmethyl-metabolite, and two of its achiral metabolites, rabeprazole-thioether and rabeprazole-sulfone in human plasma. No significant differences in the pharmacokinetic profile of rabeprazole enantiomers were found after the oral administration of racemic rabeprazole to renal transplant patients. The enantiomers of desmethyl-rabeprazole were not detected in the plasma samples. The applicability of the same analytical conditions on both PPI samples can be explained by their similar structural and physico-chemical properties. However, the ability of the method to separate at least two enantiomeric pairs and two or three (including the I.S.) achiral, or single enantiomeric compounds, revealed that Chiral-CD-Ph columns show good chemoselectivity. It is worth mentioning that in both cases, relatively long analysis times were obtained (40 and 60 min, respectively), and unfortunately, no details regarding the optimization of separation conditions are described in the papers [25,26].

An NP method for the determination of an enantiomeric impurity and a chemically related impurity of the antimigraine agent zolmitriptan has been developed by Srinivasu et al. Three different chromatographic columns were evaluated, Chiralcel OD-H, Chiralpak AD-H, and Chiralcel OJ-H. It is noteworthy that the columns were screened only with a single eluent type (*n*-hexane:isopropanol, 75:25, *v*/*v*) and the mobile phase composition was optimized after the column selection to obtain better peak shapes. The optimum enantioseparation conditions consisted of a mobile phase composed of *n*-hexane:isopropanol:methanol:diethylamine in the ratio (75:10:15:0.1, *v*/*v*/*v*/*v*) on a Chiralpak AD-H column. The validated method was successfully applied to the analysis of the pharmaceutical formulation. The analysis time was above 20 min; however, the first two peaks (zolmitriptan enantiomers) were eluted in 10 min with very good resolution. With the further optimization of the separation conditions, less retention of the third compound and a shorter analysis time may be achieved [27].

In another work by Sellers et al., a Chiralpak OD-H column with an NP mobile phase was applied for the separation of the enantiomeric and positional isomeric impurities of atomoxetine, as well as the enantiomers of the desmethyl-analog impurity of the drug. The separation conditions were thoroughly optimized in terms of their mobile phase composition including the acidic and basic additives and the alcoholic modifier to enhance the selectivity and the reproducibility of the separation. The validated method can separate six analytes within 20 min. In addition to the optimized and validated method, the study describes the development of an RP-HPLC method using sulfated-β-CD as a chiral mobile phase additive and a CE method using the single isomer heptakis-6-sulfato-β-CD as a CS. Due to the successful application of the initial NP method, the latter two were not optimized nor validated [28].

The direct separation of the anticonvulsant drug eslicarbazepine acetate and its metabolites, *S*-licarbazepine, *R*-licarbazepine, and oxcarbazepine, was reported by Alves et al. using a CD-based CSP and RP conditions, applying an isocratic elution with water:methanol (88:12, *v*/*v*) as the mobile phase. The method allowed for the simultaneous quantitation of four analytes in the presence of I.S. and proved to be applicable to the study of the stereoselective disposition of the drug in mouse plasma and brain, liver, and kidney tissue homogenates. Unfortunately, the paper does not describe the details of method development and optimization; however, the method was later validated and applied for determinations from human plasma [29,30].

A dimethyl β-CD-based CSP was employed for the simultaneous separation of the antidepressant drug sertraline and eight of its impurities. Among the impurities, three stereoisomers of sertraline, two monochlorinated enantiomeric pairs (para- and meta-substituted forms), and the derivative without chlorine substitution were analyzed under RP conditions. Method optimization involved the study of the effect of the buffer type and pH, the type of organic modifier used, and the column temperature on the separation. The validated method was capable of separating all of the nine studied compounds. The presence and the position of the chlorine substitution of the analytes had an important effect on the elution order; therefore, the authors hypothesized that the chlorine atom may interact through H-bonding with the β-CD [31].

An NP method capable of the stereoselective quantification of lansoprazole in the presence of four of its related impurities was reported by Cirilli et al. Enantioseparation was achieved using an amylose-based Chiralpak IA CSP and a mobile phase consisting of methyl-tert-butyl ether:ethyl acetate:ethanol:diethylamine 60:40:5:0.1 (*v*/*v*/*v*/*v*). Lansoprazole enantiomers were separated with a high enantioselectivity factor and resolution values, respectively. The pure enantiomers of lansoprazole at the mg scale were obtained by semipreparative HPLC with very good yields on the same type of CSP. Interestingly, impurity D of the drug did not elute under the separation conditions. The LOQ for each lansoprazole enantiomer was 0.22 μg/mL. The validated method was based on the observations made during a previously published study, where the same CSP was used under NP, PO, and RP conditions to study its enantioreseparation ability towards different drugs from the class of PPIs [32,33].

The enantiomeric separation of omeprazole in the presence of its related chiral impurities was evaluated using amylose-based Chiralpak IA CSP under PO and NP conditions. The semipreparative scale separation of the drug and its related chiral compounds was also applied. The absolute configuration of the isolated enantiomers was determined by circular dichroism spectroscopy. The validated method was successfully applied for the chiral purity control of esomeprazole in pharmaceutical formulation [34].

A new bioanalytical method using a teicoplanin-based CSP under RP conditions was developed for the stereoselective determination of bambuterol, a sympathomimetic bronchodilator and its active metabolite terbutaline in rat plasma using MS detection. The method development revealed that the chiral separation of the analytes could only be achieved when using methanol as an organic component of the eluent, while isopropanol and acetonitrile provided no enantiomeric resolution. Parameters like buffer pH or concentrations had no significant effect on the resolution values; however, they influenced retention times, as well as the magnitude of MS signals. The two analytes and the I.S. were extracted from rat plasma samples by LLE and separated on the teicoplanin column with a mobile phase constituting of 20 mM ammonium acetate solution:methanol (10:90, *v*/*v*). The validated method had an LOQ value of 1 ng/mL for both compounds and was successfully adopted for a pharmacokinetic study on Winstar rats [35].

The enantiomers of omeprazole, 5-hydroxy-omeprazole, and the achiral sulfone metabolite were simultaneously determined from human plasma using an RP HPLC method by Shiohira et al. using a Chiral CD-Ph column and phosphate buffer (pH 5.0):methanol (45:55, *v*/*v*) as the mobile phase. The validated method showing good extraction recovery and LOQ values of 5 ng/mL for all analytes was employed in a pharmacokinetic study in CYP2C19-genotyped subjects for all analytes. The results demonstrate that the formation of R-5-hydroxyomeprazole has the strongest connection with the CYP2C19 genotype [36].

The retention behavior of the chiral sulfoxides albendazole and fenbendazole and their achiral in vivo sulfide precursors (the parent drugs) and sulfone metabolites was evaluated on four immobilized amylose-based CSPs (Chiralpak IA-3, Chiralpak ID-3, Chiralpak IE-3, Chiralpak IF-3) under organic–aqueous conditions. U-shaped retention patterns were obtained by varying the water content of the eluent in combination with acetonitrile, or ethanol, as an organic component of the mobile phase. With a pure aprotic eluent (acetonitrile), broad peak shapes and high retention times were observed, probably caused by the dominance of hydrophilic-type interactions (hydrogen bonds). After the addition of a small amount of water, a decrease in retention and sharpening of the peaks were observed, typical for the hydrophilic interaction chromatography (HILIC)-retention mechanism. Under water-rich conditions (generally above 20–40% water in the eluent), the increased retention behavior observed is driven by hydrophobic interactions typical to RP conditions. It was highlighted that the interactions involved in the HILIC separation mechanism have a non-enantioselective nature since the enantioselectivities were not significantly influenced by the addition of water to the mobile phase. Additionally, the influence of the dual retention mechanism on the chemoselectivity of the CSP was also evaluated. The authors revealed that the elution order of sulfide and sulfone derivatives was in accordance with their intrinsic hydrophilicity and the dualistic nature of the analyte/CSP interactions. The best simultaneous separations were obtained when using ethanolic:water eluent mixtures and a Chiralpak IA-3 column, with reasonable analysis times (10 min and 13 min for albendazole and fenbendazole derivatives, respectively) [37].

Chiralpak IA, IB, and IC CSPs were tested under the RP mode to develop a simultaneous method for the determination of the enantiomeric and organic impurities of R-rabeprazole. After column selection (Chiralpak IC with cellulose tris-(3,5-dichlorophenylcarbamate) CSP), further method optimization consisted of the proper selection of an optimum buffer composition and column temperature. While the isocratic mode was applied during the scouting experiments, this was switched for a gradient elution to reduce the analysis time and improve the shapes of the later-eluting peaks. Additionally, the chemical structures of two unknown impurities of the drug were elucidated based on their ESI-MS/MS fragmentation patterns. The final, validated LC-UV method could determine the enantiomeric and three other organic impurities of rabeprazole at 0.02–0.03% concentrations relative to rabeprazole [38].

A PO method using a cellulose tris(3-chloro-4-methylphenylcarbamate) CSP was reported for the simultaneous determination of the chiral antiepileptic prodrug eslicarbazepine acetate and its major metabolites eslicarbazepine, R-licarbazepine, and the achiral oxcarbazepine. Method optimization was carried out by studying the effect of the methanol content of the mobile phase on the retention behavior of the analytes. A significant influence of the methanol percentage on both the resolution and retention of the analytes was found. The optimized mobile phase consisted of acetonitrile:methanol:acetic acid:diethylamine (95:5:0.2:0.07, *v*/*v*/*v*/*v*). Moreover, the elution order reversal between R-licarbazepine and oxcarbazepine (two compounds with very similar structures, but not enantiomers) was observed by increasing the methanol content of the eluent mixture. The authors explained this phenomenon with a possible change in the conformation of the CSP under different mobile phase compositions. The validated method proved to be applicable for the in vitro study of the stereoselective metabolism of eslicarbazepine acetate [39].

The chromatographic behavior of the enantiomers of bicalutamide, a non-steroidal antiandrogen used for the treatment of prostate cancer, and their chiral impurities was evaluated by Sadutto et al. using an immobilized amylose-based Chiralpak IA CSP. Due to the immobilized nature of the CSP, both standard *n*-hexane:ethanol and ethyl acetate-containing “non-standard” NP eluents could be used. Non-linear van’t Hoff plots were obtained in the case of the “non-standard” mobile phase (*n*-hexane:ethyl acetate:ethanol 100:30:5, *v*/*v*/*v*), while the eluent without ethyl acetate showed linear plots. The simultaneous separation of the four stereoisomers of bicalutamide-sulfoxide was achieved using the standard mobile phase. The analytical and semi-preparative HPLC resolution of bicalutamide chiral impurities, as well as their empiric absolute configuration assignment using the circular dichroism correlation approach, are also described in the study [40].

**Figure 1:**
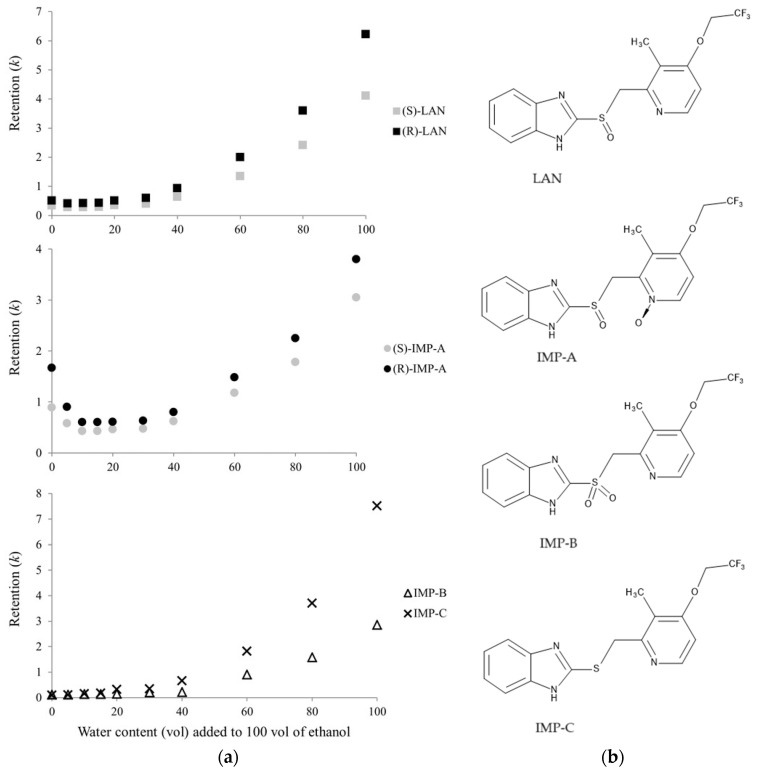
(**a**) Plots of the retention of lansoprazole (LAN), IMP-A, IMP-B, and IMP-C as a function of the water content in the ethanol/aqueous mobile phase (column Chiralpak IC-3 (100 mm × 4.6 mm); UV detection 280 nm; flow rate 1.0 mL/min; column temperature 25 °C). Reprinted from Ferretti et al. [41] with permission from Wiley; (**b**) Chemical structures of lansoprazole and its impurities A, B, and C.

In another study published by Ferretti et al., Chiralpak IC-3 CSP was employed in combination with the ethanol:water mixture as the eluent to develop a method for the enantiomeric determination of lansoprazole in the presence of its related impurities. The impact of the water content of the mobile phase on the retention behavior of the analytes was investigated. Similarly, U-shaped retention profiles were observed in the case of the enantiomers of lansoprazole and those of its N-oxide derivative (impurity A). In the latter case, the transition from the HILIC to RP mode was more evident, which can be explained by the higher hydrophilic character of the impurity resulting in more pronounced interactions with the polar sites of the CSP responsible for the HILIC interactions. In the case of the achiral sulfone and sulfide derivatives (impurity B and impurity C, respectively) RP retention profiles were obtained. The authors concluded, that in the case of impurity B, despite the polar character of the sulfone group, the interaction of this with the specific sites of the CSP is hindered, while in the case of impurity C, the lower hydrophilicity of the compound is responsible for the lack of HILIC interactions. On the other hand, after the establishment of the final RP separation conditions, the elution order of the different lansoprazole derivatives (sulfide, sulfoxide, and sulfone) showed a good correlation with their intrinsic hydrophilicity. The enantiomeric elution order was established by isolating the pure enantiomers of the two chiral analytes by an NP method and applying circular dichroism correlation. Figure 1 shows the above-mentioned retention profiles of the enantiomers of lansoprazole, the enantiomers of Impurity A, and two achiral impurities (Impurity B and Impurity C) [41].

A validated method capable of the determination of the enantiomeric purity of esomeprazole in the presence of its related compounds was also reported by Cirrilli et al. The RP approach was based on the optimized conditions for the chiral separation of omeprazole, performed in a previous work [33]. A comparison of the described method with the compendial one, from the European Pharmacopoeia (Eur Ph) was performed. The latter one employs a Chiral-AGP (100 mm × 4.0 mm, 5 μm) column with a mobile phase containing a mixture of acetonitrile:phosphate buffer (pH 6.0) (13:87, *v*/*v*) for the enantiomeric purity analysis of the drug. The newly developed method can determine *R*-omeprazole without any interferences from the related impurities, while when using the official method, an overlapping of the peaks of *R*-omeprazole (impurity F) and impurity A can be observed [42].

A RP method using cellulose-based Chiralcel OJ-RH CSP was developed by Ferretti et al. for the simultaneous separation of the antiplatelet drug clopidogrel and its chiral impurities, and compared with the method reported in the United States Pharmacopoeia (USP) monography, which employs an Ultron ES-OVM column. The *S*-enantiomer was the baseline, resolved from its *R* impurity using a mobile phase of methanol:water (100:15, *v*/*v*), with no interference from the other two possible chiral impurities. The novel method provides a favorable elution order, with all the impurity peaks appearing before the peak of clopidogrel on the chromatogram, while in the chromatogram obtained under the conditions described in the USP method, the peak of the second eluting (*R*)-impurity B enantiomer is masked by the *S*-clopidogrel peak. Additionally, the enantiomers of Impurity-B were isolated by semipreparative NP-HPLC, and their configurational stability was determined [43].

Guo et al. explored mobile phase parameters acceptable for chiral analysis using ESI LC-MS, with glycopeptide antibiotic vancomycin as the CSP. In this, 2.7 µm of vancomycin-containing superficially porous particle-based CSPs were compared to its 5 µm fully porous particle-based variant under ESI-MS detection-friendly eluent conditions. Column efficiency, selectivity, and analysis time towards 22 basic chiral drugs were evaluated. The superficially porous particle-based CSPs provided better results in all cases and were applied to the stress degradation study of citalopram enantiomers. The results demonstrated that the superficially porous particles column provides higher separation efficiencies, better sensitivity, and shorter analysis times. The enantiomers of citalopram and two of its degradation products could be separated and identified based on their MS characteristics, using 5 mM ammonium acetate buffer:methanol (10:90, *v*/*v*) as the mobile phase [44].

Seven polysaccharide-type CSPs from the Lux series were evaluated for the separation of the phenothiazine neuroleptic drugs dextromepromazine, levomepromazine sulfoxide, and 2-methoxyphenothiazine in levomepromazine samples in PO mode. Lux Cellulose-3 (cellulose tris(4-methylbenzoate)) and Lux Amylose-1 (amylose tris(3,5-dimethylphenylcarbamate)) columns provided a proper chiral recognition capability towards mepromazine enantiomers. Lux Celllose-3 CSP was chosen based on the better resolution values obtained between enantiomers, while showing good chemoselectivity as well. However, when using 0.1% diethylamine containing methanol as an eluent, the levomepromazine sulfoxide eluted very close to the dead volume and different approaches applied, such as the addition of water or the change of methanol to acetonitrile or ethanol, did not provide any improvement. The LOD values were in the range of 0.002 to 0.005 µg/mL and the method was successfully applied for the analysis of pharmaceutical products [45].

A validated method for the separation of guaifenesin enantiomers and ambroxol was reported using a cellulose-(3,5-dimethylphenylcarbamate) CSP under RP conditions by a gradient elution in 8 min. The author claimed the necessity of green methods and the study contains a green assessment of the developed method. The gradient profile used for the separation of the racemic guaiafenesin and ambroxol binary mixture was optimized using an experimental design. Using Lux Cellulose-1 as a chiral stationary phase and ethanol:water as a mobile phase, the chromatographic technique was accomplished in 6 min. with a linear gradient elution of 20% to 70% ethanol. However, from a practical point of view, a simpler isocratic method might be developed with proper optimization. If other green organic solvents, such as isopropanol, were screened in combination with the five CSPs tested, or the effect of other parameters, such as column temperature or acidic/basic additives, was studied, a rapid and effective separation method might be obtained without the application of a gradient elution [46].

Since the biological degradation processes and the ecotoxicity of chiral pharmaceuticals are generally stereoselective, the chiral determination of drugs in environmental samples has become a major problem [47]. A selective LC-MS/MS method was reported by Ma et al. for the enantioselective quantitation of three non-steroidal anti-inflammatory drugs (flurbiprofen, ibuprofen, and naproxen) in surface water samples using Chiralpak AD-RH CSP under RP conditions. An ESI source, operating in a negative mode, and MRM detection were applied. The method was optimized in terms of the type and proportion of the organic modifier of the eluent, the effect of the column temperature, the buffer type, and the pH to obtain a good chiral resolution, short analysis time, and adequate MS response. Acetonitrile provided better results than methanol as an organic component of the eluent, while the pH of the buffer seemed to be the main influencing parameter on both the retention and the MS signal intensity, as could be expected in the case of organic acid analytes. The LOQ values of the method were in the range of 1.1–37.1 ng/L. The validated method was applied to the analysis of environmental samples. Ibuprofen and naproxen were detected in the majority of the 24 sampling sites, while flurbiprofen was only in several cases. Both ibuprofen and naproxen showed an excess of the *S*-enantiomer in different surface water matrices; however, the enantiomeric proportion presented significant variations depending on the sampling site and the sampling period [48].

The simultaneous determination of the enantiomeric impurity and four other organic impurities of sertraline, a selective serotonin reuptake inhibitor (SSRI), was reported by Rosetti et al. using an RP method on an amylose tris(3-chloro-5-methylphenylcarbamate) CSP in a single chromatographic run. Method optimization involved the evaluation of the effect of a mobile phase composition and column temperature on the separation. The validated method enables the baseline separation of all studied compounds in 15 min. and can be applied for determinations from a pharmaceutical formulation [49]. 

Two chiral columns, the coated Chiralpak AS-H, and the immobilized Chiralpak IH-3, having the same CS, namely amylose tris-[(*S*)-α-methylbenzylcarbamate], have been evaluated and compared for the chiral separation of α-lipoic acid and α-dihydrolipoic acid under NP conditions. The immobilized CSP permitted the use of non-standard NP eluent types, containing organic solvents such as dichloromethane and ethyl acetate, tetrahydrofuran, or 2-methyltetrahydrofuran, in addition to the classical alkane:alcohol mobile phase composition. By applying these new eluent types, in several cases, a better enantiomeric resolution could be obtained; however, the effect of the difference in the particle size of the two columns was not evaluated. The thermodynamic study revealed that the enantiomeric separation process was enthalpically driven for both compounds. The simultaneous chiral separation of both acids was achieved in 25 min. using the Chiralpak AS-H column. An analytical-size Chiralpak IH-3 column was used to extract enantiomers, which were subjected to chiroptical measurements. An interdisciplinary process based on a comparison of the estimated and experimental chiroptical properties identified the separated enantiomers’ absolute configuration. It is noteworthy, that the comparison of the column performances under PO and RP conditions could have provided further valuable information and a more ecological separation method [50].

An RP method using a cellulose-methylbenzoate-based CSP and a gradient elution was developed by Tóth et al. for the simultaneous quantification of the enantiomeric and organic impurities of dapoxetine, an SSRI used to treat premature ejaculation. The screening phase involved the testing of three amylose- and four cellulose-based CSPs from the Lux series under PO mode using 0.1% (*v*/*v*) diethylamine containing methanol, isopropanol, or acetonitrile as the eluents. After column selection, the method was further optimized in terms of eluent composition, including the type and concentration of organic modifiers and the acidic/basic additives. To ensure proper resolution values for each peak pair and an acceptable analysis time, a gradient elution in combination with flow programming was applied. The best results were obtained on a Lux Cellulose-3 column with the ethanol-based mobile phase, and using the optimized conditions, baseline separations were obtained for all substances within 30 min. Although impurity-1 and impurity-2 eluted relatively close to the dead volume, this fact did not affect their quantitative determination. The final method was able to determine the studied impurities in the concentration range of 0.05–0.16%, relative to the concentration of dapoxetine. The application of the method involved the analysis of both pharmacy- and Internet-purchased samples. It is noteworthy that the products purchased from the Internet showed low quality, containing elevated levels of the enantiomeric impurity [51].

Seven polysaccharide-based CSPs were tested under PO conditions using 0.1% diethylamine containing methanol, isopropanol, or acetonitrile as the mobile phase to achieve the simultaneous separation of the heart-rate-lowering drug ivabradine, its enantiomeric impurity, and four of its related compounds. Method optimization was performed using experimental design methodology in terms of the concentration of diethylamine in the mobile phase, eluent composition, flow rate, and column temperature. During the screening studies, the best results were obtained on a Lux Cellulose-2 (cellulose tris(3-chloro-4-methylphenylcarbamate) column with methanol, and a favorable elution order (all impurities eluting before the main peak) was obtained with a baseline separation for all peak pairs in 25 min. The method was successfully applied for the analysis of the ivabradine-containing pharmaceutical formulation [52].

The separation capability in terms of the enantio-, diastereo-, and chemoselectivity of Chiralpak IA-3 CSP was evaluated by Rosetti et al. on paroxetine, an SSRI antidepressant, and its related substances. Paroxetine possesses two stereogenic centers, and therefore, four stereoisomers can exist, but it is used in therapy in the form of a single isomer. In the Eur. Ph. Monograph, Impurity-D is the enantiomeric impurity, while Impurity-E represents the diasteremic pair of enantiomers. Different types of RP eluents were tested, containing acetonitrile, ethanol, or methanol as an organic modifier to achieve simultaneous enantio- and diastereoselectivity. Both alcoholic-type modifiers proved to be well-suited for this purpose. The thermodynamic investigation showed that the separation of diastereomers was entropically driven, while the separation of enantiomers was an enthalpically driven process. Although the mobile phase containing methanol proved to be the most favorable in terms of enantio- and diastereoselectivity, no appropriate chemoselectivity could be achieved with it. The optimized method, based on a mixture ethanol:water:diethylamine (80:20:0.1, *v*/*v*/*v*) as a mobile phase could separate the enantiomers of paroxetine from each impurity examined in the study [53].

The efficiency of two amylose tris(3,5-dimethylphenylcarbamate)-based columns differing in their length (5 and 10 cm) and the combination of the same type of column with an achiral one (5 cm biphenyl column connected by a zero dead volume connector to a 5-cm chiral column) was compared and evaluated by Camilo et al. with emphasis on the selectivity and analysis time. The racemic forms of four non-steroidal anti-inflammatory drugs (flurbiprofen, ibuprofen, ketoprofen, and naproxen) were used as model compounds. Three of the four enantiomeric pairs could be resolved under the applied conditions, namely, ibuprofen, naproxen, and flurbiprofen. Both isocratic and step-gradient approaches were studied, and the findings were compared to evaluate the advantages and disadvantages of each strategy, with a focus on the resolution and analysis time. The obtained results revealed that column-coupling could provide shorter analysis times and better resolution values in some cases. Typically, the combination of an achiral column with a chiral one increased the chemoselectivity of the method, but enantioselectivities were higher when using a chiral column with the same length [54].

A method suitable for the quality control of escitalopram was reported by Szabó et al. for the determination of the enantiomeric impurity and three potential synthesis-related chemical impurities of the drug. The scouting step of the method involved the evaluation of three amylose-based and four cellulose-based CSPs under PO and RP conditions. After the selection of the column and the type of the mobile phase, a diethylamine containing a mixture of acetonitrile and water, method optimization was performed involving the study of the temperature, flow rate, and eluent composition. Plotting the citalopram enantiomers’ retention factors against the binary mobile phases’ water concentration on the Lux Cellulose-1 column produced a U-shaped retention pattern, typical to the HILIC-RP dual behavior of such types of CSPs. The chiral separation mechanism proved to be an enthalpically driven process both in PO and RP conditions. The validated method could determine the examined impurities at a 0.05–0.1% concentration relative to an escitalopram in 14 min and proved to be applicable to real pharmaceutical samples [55].

Two new approaches, an NP and an RP HPLC method, were developed by Cantatore et al. for the simultaneous determination of the enantiomeric and organic impurities of escitalopram, an SSRI antidepressant on a cellulose-based Chiralcel OJ-H CSP. In the pharmacopeial monographs from both Eur Ph. and USP, the chiral impurities of escitalopram are reported in their racemic form and not with an *S*-configuration. Therefore, enantioenriched forms of impurities were collected using the same analytical column and their absolute configuration was assigned based on their electronic circular dichroism characteristics. No organic impurity showed interference with the citalopram enantiomers. Moreover, the enantiomers of all chiral impurities except impurity C could be separated with the applied NP and RP methods. The LOQ for the *S*- and *R*-enantiomers were 4.5 and 3.8 μg mL^−1^, respectively [56].

Four cellulose-based CSPs (Chiralpak IB-3, Chiralpak IJ-3, Chirapak IC, and Lux Cellulose-2) were evaluated under RP conditions using 0.05% trifluoroacetic acid (TFA) in combination with different organic modifiers (acetonitrile, ethanol, and methanol) as mobile phases for the chiral separation of rosuvastatin, a competitive inhibitor of the enzyme HMG-CoA reductase. Under the applied conditions, only the Lux-Cellulose-2 column showed proper enantioselectivity with mixtures containing an organic modifier (ethanol, methanol, or acetonitrile) and TFA 0.05% in an aqueous solution used as the mobile phases. However, the method showed good selectivity and the high retention of impurity-A caused a long analysis time (above 80 min). Therefore, gradient elution was applied to obtain a shorter separation. When compared to the isocratic HPLC method officinal in Eur. Ph. for rosuvastatin, the gradient elution method provided shorter analysis times and better chemo- and enantio-selectivity. The LOD and LOQ of the enantiomeric impurity were 0.05 and 0.15 µg/mL. The method after its partial validation was applied to the analysis of pharmaceutical products [57].

**Figure 2:**
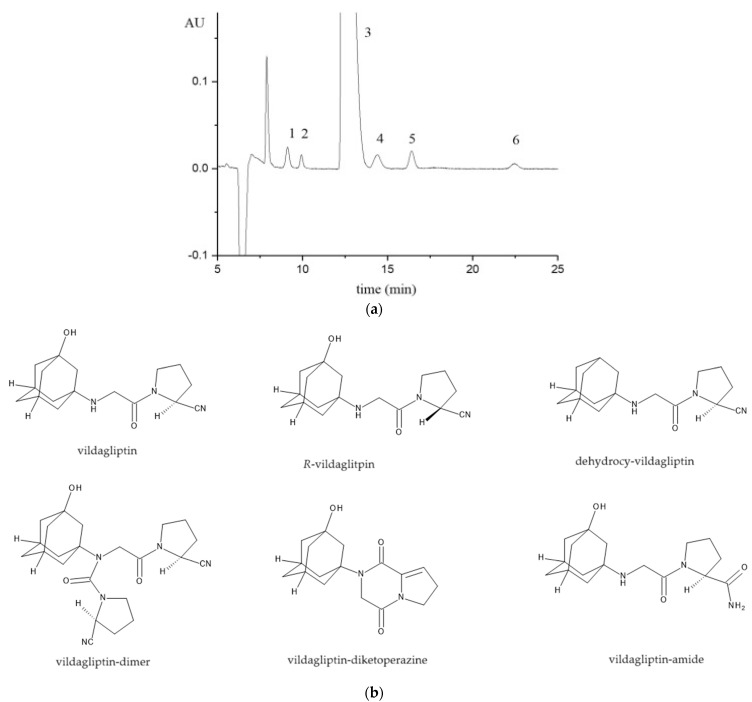
(**a**) Chromatogram of vildagliptin sample spiked with 0.1% of each impurity under optimized conditions (CSP–Lux Cellulose-2, column temperature: 45 °C, mobile phase: methanol:water:diethylamine 80:20:0.2 (*v*/*v*/*v*), flow rate: 0.45 mL/min, UV detection at 215 nm). (1—vildagliptin-amide, 2—*R*-vildagliptin, 3—vildagliptin, 4—vildagliptin-dimer, 5—vildagliptin-diketopiperazine, 6—dehydroxy-vildagliptin). Reprinted from Papp et al. [58] with permission from Elsevier; (**b**) Chemical structure of vildagliptin and its impurities.

Recently, a new RP-HPLC method was reported by our research group for the simultaneous determination of the oral antidiabetic drug vildagliptin enantiomeric impurity and four other achiral-related compounds of the drug. Five polysaccharide CSPs were evaluated (Lux Amylose-1, Lux Amylose-2, Lux-Cellulose-1, Lux Cellulose-2, and Lux-Cellulose-3) in PO mode, with the mobile phase consisting of neat methanol, ethanol, isopropanol, or acetonitrile containing 0,1% diethylamine. After column selection, based on its chiral recognition capability, different aqueous–organic eluents were tested to achieve the simultaneous separation of all analytes of interest. The Lux-Cellulose-2 column was found to provide the best chiral resolution for vildagliptin enantiomers. Further studies were carried out with various aqueous–organic mobile phases to achieve the simultaneous chiral–achiral separation of the selected compounds. Method optimization consisted of an experimental design methodology in two steps: a screening design followed by an optimization one. The optimal conditions enabled the determination of all impurities with an at least 0.1% concentration relative to vildagliptin in 25 min [58]. A representative chromatogram obtained under optimized conditions is shown in Figure 2.

An overview of the above-presented separation methods is summarized in Table 1. The studies are listed chronologically. This chronological organization helps understand the progression of research, the emergence of trends or patterns, and the evolution of theories or methodologies.

## 3. Discussion

### 3.1. Different CSP Types and Their Applications in Simultaneous Chiral–Achiral Separations

Polysaccharide-based CSPs are the most successful and commonly used CSP class in chiral chromatography, accounting for approximately 90% of all documented chiral HPLC procedures. Their first applications date back to the 1970s [10,59], while their applicability in HPLC as phenylcarbamate derivatives coated on silica particles became possible due to the work of Okamoto et al. in 1984 [60]. The most common polysaccharide-based CSPs are cellulose and amylose carbamates and esters; however, other derivatives, such as those of chitin and chitosan, were also successfully applied. Their versatility regarding the wide range of compatible solvents makes their use in different separation modes (NP, PO, HILIC, and RP) possible. Moreover, the chemically immobilized CSPs can be used with “non-standard” solvents (ex. acetone, chloroform, dichloromethane, ethyl acetate, tetrahydrofuran, etc.); however, the immobilization can influence their chiral selectivity [61,62,63]. Their specific structural properties, such as the conformational chirality of the helical carbohydrate backbone, the stereogenic centers of the monosaccharide units, as well as the functional groups of the ester and phenylcarbamate substituents attached to the polysaccharide chain, provide the formation of different types of intermolecular interactions (hydrogen bonds, dipole–dipole, π–π, inclusion complexation, etc.) involved in the chiral recognition mechanism of the CSPs, making the chiral separation of a large variety of substances of pharmaceutical interest possible [64,65].

Polysaccharide CSPs proved to be capable of the simultaneous separation of enantiomers and achiral-related compounds as well as drug metabolites for determinations from pharmaceutical and biological samples. Their applications not only include classical NP and RP chromatography, but they were successfully employed in PO and HILIC modes as well. The effect of the eluent type used on the enantiomeric recognition process is hard to predict since enantioselective analyte–CS interactions have a very complex nature, involving not only the different intermolecular forces responsible for the formation of enantiomer–CS complexes, but also the possible conformational changes of the carbohydrate structure in the function of the eluent composition. Other parameters, such as the effect of the column temperature, the concentration of acidic/basic additives, etc., must be considered as well [10]. In addition, the very interesting HILIC behavior of polysaccharide CSPs in aqueous–organic mobile phases has a non-enantioselective nature, with the enantioselectivity of the CSPs remaining the same in many cases when changing the water content of the eluent [37,41,66,67]. This phenomenon enables the optimization of the separation of analytes which have different chemical structures, based on their hydrophilic/hydrophobic character, without a significant change in the chiral resolution.

A variety of polysaccharide CSPs were applied to simultaneous chiral–achiral separations showing their versatility. The most often applied ones are the tris-(3,5-dimethylphenylcarbamate) derivatives of cellulose [16,17,18,21,24,28,46,55] and amylose [11,27,32,34,37,41,48,53], but other derivatives, such as amylose tris-(3-chlorophenylcarbamate) [42], cellulose tris-(3,5-dichlorophenylcarbamate) [22,38,41], cellulose tris(3-chloro-4-methylphenylcarbamate) [39,52,57,58], cellulose tris(4-methylbenzoate)-based [43,45,51,53], amylose tris(3-chloro-5-methylphenylcarbamate) [40], amylose tris[(*S*)-α-methylbenzylcarbamate], are also used [50]. In several cases, gradient elution was applied in combination with these columns [18,38,46,57].

Protein-type CSPs can interact stereoselectively with many chiral drugs due to their complex structure, involving a high variety of different functional groups. Their chiral recognition mechanism is mainly based on the formation of hydrogen bonds and π–π, dipole–dipole, and ionic interactions. However, their limitations, such as their sensitivity to pH, ionic strength, and organic constituents of the eluent, contributed to the decrease in their popularity over the past two decades, in comparison to the novel CSPs with fewer stability issues. Another disadvantage of protein CSPs is their insufficient loading capacity for preparative applications. The most common representatives include human serum albumin, bovine serum albumin, α_1_-acid glycoprotein, ovomucoid, and cellobiohydrolase CSPs [68,69]. Only several types of protein CSPs were used in simultaneous enantio- and chemoselective separations, such as CBH- [15,23] and AGP-based [12,14] columns. These were employed in the enantiomeric determination of several drugs and their main metabolites to assess the stereoselectivity of their metabolism. Although protein CSPs can be applied to analytes from different classes with generally good enantioselectivity, their chemoselective properties in many cases are not sufficient, and only two or three structurally different compounds can be separated in the same chromatographic run.

Immobilized β-CD-based CSPs appeared in the 1980s and still represent a field of great research interest. The chiral recognition mechanism of β-CD derivatives is mainly based on the inclusion complexation of molecular moieties such as aromatic cycles or other hydrophobic groups into the hydrophobic CD-cavity and the formation of secondary interactions, such as hydrogen bonds and dipole–dipole or ionic interactions (in the case of ionizable CDs) [70,71]. The formation of non-inclusion (surface-type) complexes is also possible, especially in NP and PO modes [72,73]. Different CD derivatives, such as native β-CD, phenylcarbamate-β-CD, and chlor-, or methyl-substituted phenylcarbamate-β-CDs are the most frequently applied forms of CSPs [74], prepared predominantly by different immobilization techniques, such as by ether or urea linkages [75,76,77], click-chemistry [78], etc. Besides their high versatility as chiral selectors, CDs already proved their chemoselective potential in electromigration techniques [79,80,81,82], as well as CSPs in the analysis of achiral compounds [83,84,85]. Until now, native β-CD [20,30] as well as its dimethylated [31] and phenylcarbamated [25,26,36] derivative CSPs have been applied in simultaneous chiral–achiral separations, including the bioanalytical determination of drug enantiomers and their metabolites, as well as methods developed for the quality control of pharmaceuticals.

Glycopeptide macrocyclic antibiotics-based CSPs appeared in the 1990s and represent a tool of high versatility in chiral HPLC due to their complex interaction capabilities, such as electrostatic, hydrogen bonding, hydrophobic, dipole–dipole and π–π interactions, and steric repulsion. Moreover, their multimodal nature allows for their application in RP, NP, PO, and polar ionic (PI) modes [86]. The multiple types of functional groups present in the structure of glycopeptide antibiotics offer a complex pattern of chiral recognition capabilities in the function of the applied eluent type and the analyte nature. Therefore, a high variety of enantiomeric pairs can be resolved by using them. Additionally, due to their similar enantioselectivity and higher stability and compatibility with different eluent types, in many cases, they replaced the protein-based CPSs [87,88]. Only a few enantio- and chemoselective methods have been published using teicoplanin [19,35] or vancomycin-based [44] CSPs for bioanalytical purposes or as being suitable for the quality control of chiral drugs. The developed methods were able to separate several chiral drugs and their metabolites, as well as the enantiomers of citalopram and their degradation products. In addition, due to the similar structural and physicochemical properties of glycopeptides, good chemoselectivity can be expected for other antibiotic CSPs as well.

### 3.2. Chemoselective Separation Mechanisms Involved in Achiral Separations on CSPs

The chiral recognition mechanisms underlying the retention behavior of enantiomers on different types of CSPs have been intensively investigated; however, information regarding the behavior of achiral compounds on CSPs is deficient and many times, conclusions are drawn based on the observations gained during chiral analysis. As stated in a recent work by Scriba et al., “it should be kept in mind that very often the selectors also display chemoselectivity because binding thermodynamics also differ for structurally related or unrelated compounds. Thus, chiral selectors may enable the simultaneous separation of stereoisomers and other compounds.” [89]. This assumption is supported by theoretical considerations as well as by experimental results.

**Figure 3:**
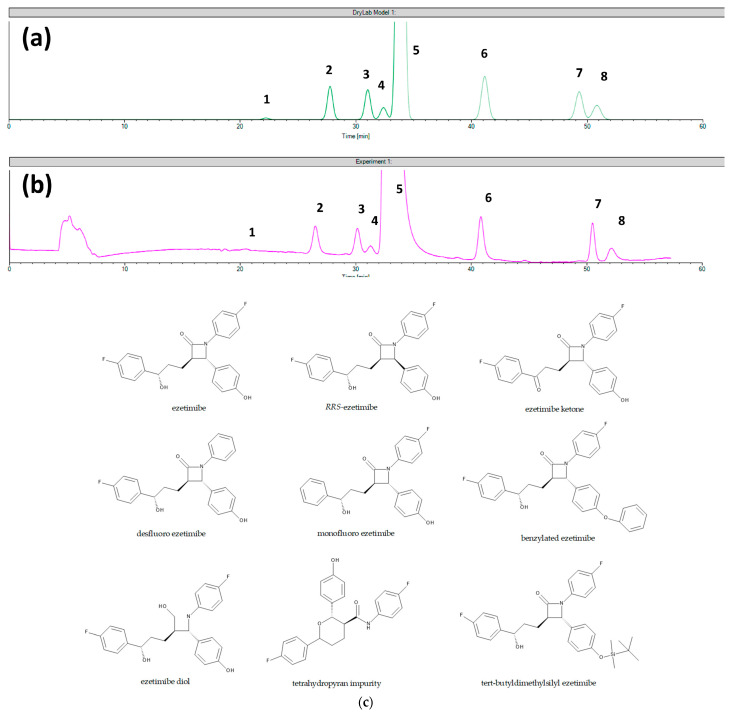
The comparison of (**a**) the virtual chromatogram generated by the DryLab software (version 4.5) and (**b**) the experimental chromatogram obtained in the case of setpoint 1 tested for Chiral CD-Ph column (1—ezetimibe diol; 2—THP (tetrahydropyran) compound; 3—RRS ezetimibe; 4—monofluoro ezetimibe; 5—ezetimibe API; 6—ezetimibe ketone; 7—TBDMS (tert-butyldimethylsilyl) ketone; 8—benzylated ezetimibe). Reprinted from Ferencz et al. [90]; (**c**) Chemical structure of ezetimibe and its impurities.

**Figure 4:**
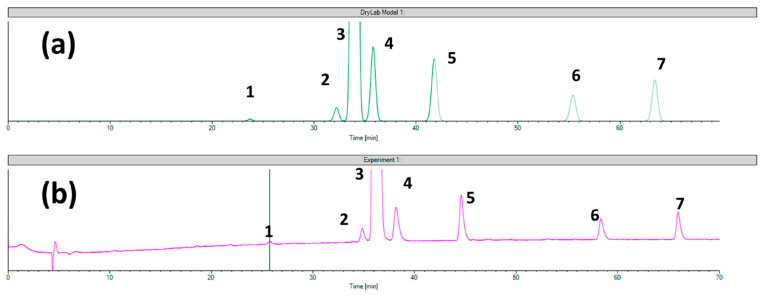
The comparison of (**a**) virtual chromatogram generated by the DryLab software (version 4.5) and (**b**) the experimental chromatogram obtained in case of setpoint 1 tested for Chiralcel OD column (1—ezetimibe diol; 2—desfluoro ezetimibe; 3—ezetimibe API; 4—THP (tetrahydropyran) compound; 5—ezetimibe ketone; 6—TBDMS (tert-butyldimethylsilyl) ketone; and 7—benzylated ezetimibe). From Ferencz et al. [90].

In the first approach, the chemoselectivity of CSPs can be derived from non-stereoselective analyte–selector interactions, depending on the physicochemical and structural properties of both the CS and analyte, but the contribution of residual silanol groups is also possible. The experimental findings show that the achiral separation mechanism of several CSPs shows a good correlation to the intrinsic hydrophilicity of the analyte molecules [37,41]. In a recent study, computer-assisted mechanistic modeling showed a very good correlation between the predicted and experimental data in terms of the retention times and resolution values of ezetimibe and its related compounds obtained in the case of the Chiral CD-Ph column. In addition, the same software showed less prediction accuracy in the case of the Chiralcel OD CSP towards the same analytes. The different behaviors of the polysaccharide CSP can be explained by its distinct separation mechanism, involving other types of interactions than those present in a classical achiral RP system [90]. Virtual and experimental chromatograms obtained for ezetimibe and its related substances on Chiral CD-Ph and Chiralcel OD columns are presented in Figure 3 and Figure 4, respectively. The verification of the predictability of chiral separations using the same software was rather unsuccessful. The teicoplanin and ristocetin A- and amylose tris (3,5-dimethylphenylcarbamate)-based CSPs did not show a specific RP retention behavior, probably due to the importance of the hydrogen bond interactions in their separation mechanism. Interestingly, the vancomycin-based CSP showed a typical RP retention mechanism with good predictability of its enantiomeric selectivity [91,92]. Similarly, the hydroxypropyl-modified β-cyclodextrin CSP showed good predictability in the separation of atropisomers [93]. As a summary of these observations, the mechanism of both enantiomeric and chemoselectivities under RP conditions of CD-based CSPs seems to be a similar process to achiral systems described by the solvophobic theory [94,95], while the application of chromatographic modeling can be a very useful tool in method development by shortening the whole process. In the case of polysaccharide and glycopeptide CSPs, the additional interactions provide a different retention mechanism, especially for enantiomeric separations; therefore, method development should indispensably contain a trial-and-error phase and the modeling can be used for the fine-tuning of the separation conditions.

### 3.3. Future Perspectives

It is worth noting that experimental design-based method optimization was successfully applied in several cases in combined enantio- and chemoselective separations, proving that statistical approaches based on the building of empirical models still can be employed independently from the nature of the retention mechanism [46,52,58]. Quality by design-compliant method development, as an almost mandatory approach in the current pharmaceutical analysis, including the application of statistical tools offered by experimental design methodologies, will probably have an accentuated presence soon in single-CSP-based chiral–achiral separations as well [96,97].

The necessity of developing new methods showing both enantio- and chemoselectivity is demonstrated by the fact that in several cases, the insufficient chemoselectivity of compendial methods used for the control of the enantiomeric purity of drugs was demonstrated [42,53,56]. In such cases, specific attention must be paid to avoid interferences from other related compounds that could coelute with the chiral impurity examined. Additionally, the necessity of the enhanced selectivity of methods capable of the analysis of a group of structurally similar compounds from an environmental matrix is more than evident; therefore, the publication of more simultaneous chiral–achiral assays on this topic can be expected.

The already well-known trend in achiral chromatography of reducing both column dimensions and particle sizes of stationary phases, while higher separation efficiencies are achieved, can be observed in the field of CSPs as well. The technological development in particle production and column packing procedures already has a great impact on chiral separation efficiencies and analysis times. These facts can offer further perspectives in combined chiral–achiral analyses, such as efficient column-coupling procedures, but also better chemoselective properties for the new generation of CSPs. However, to benefit from these opportunities, new-generation equipment is also needed, with increased pressure resistance and a high detection frequency [98,99,100,101].

## 4. Conclusions

Simultaneous chiral–achiral HPLC separation methods employing a single-column approach, namely the application of a CSP, have been overviewed in the current study.

CSPs employed in simultaneous chiral–achiral separation must possess a chiral recognition mechanism that allows for the efficient separation of enantiomers, while also accommodating the retention of achiral compounds. This necessitates a delicate balance between stereoselective interactions for chiral separation and non-stereoselective interactions for achiral retention. Chemoselectivity, the ability to selectively retain analytes based on their chemical properties, is paramount in simultaneous chiral–achiral separations.

Polysaccharide-based CSPs stand out as the most successful and widely utilized class in chiral chromatography as the versatility of these CSPs, particularly cellulose and amylose carbamates and esters, extends across various separation modes, allowing for their application in NP, PO, HILIC, and RP chromatography. The unique structural properties of polysaccharide CSPs, including the conformational chirality of the helical carbohydrate backbone, stereogenic centers of monosaccharide units, and functional groups of esters and phenylcarbamate substituents, contribute to diverse intermolecular interactions crucial for chiral recognition. Such attributes enable the simultaneous separation of enantiomers, achiral compounds, and drug metabolites from complex pharmaceutical and biological samples.

While protein-type CSPs exhibit stereoselectivity with various chiral drugs, their limitations in terms of sensitivity to pH, ionic strength, and organic constituents have led to a decrease in popularity. Immobilized β-CD-based CSPs offer high versatility as CSs have been successfully employed in simultaneous chiral–achiral separations, including the bioanalytical determinations of drug enantiomers and their metabolites. Glycopeptide macrocyclic antibiotics-based CSPs present a versatile tool in chiral HPLC due to their complex interaction capabilities; they offer multimodal applicability in RP, NP, PO, and PI modes. 

The chiral recognition mechanisms on different CSPs have been extensively studied, but there remains a deficiency in understanding the retention mechanism involved in the chemoselective separation processes. The chemoselectivity of CSPs may arise from non-stereoselective analyte–selector interactions, with experimental findings suggesting a correlation between achiral separation mechanisms and the intrinsic hydrophilicity of analyte molecules.

Looking ahead, quality by design-compliant method development, incorporating statistical tools, is expected to play a more prominent role in optimizing single-CSP-based chiral–achiral separations. Furthermore, technological advancements in particle production and column packing procedures hold promise for the next generation of CSPs, offering new perspectives to address current analytical challenges.

The continuous development and application of diverse CSPs in chiral chromatography offer valuable tools for addressing complex analytical challenges in pharmaceutical and environmental analyses, emphasizing the need for enhanced selectivity in simultaneous enantio- and chemoselective separations.

## Figures and Tables

**Table 1:** HPLC methods published for combined chiral–achiral separations.

Analytical Conditions	Column/CSP	Samples, Extraction Procedure	Analytes	Reference
mobile phase: *n*-hexane:isopropanol:diethylamine (90:10:0.1, *v*/*v*), flow rate: 1.0 mL/min, fluorescence detection: excitation 223 nm, without using an emission filter	Chiralpak AD (50 mm × 4.6 mm, 5 µm), CSP: amylose-tris(3,5-dimethylphenylcarbamate)	human plasma LLE	verapamil norverapamil	[11]
mobile phase: 0.01 M phosphate buffer (pH 6.65):acetonitrile (91:9, *v*/*v*), flow rate: 0.9 mL/min, fluorescence detection: excitation 227 nm, emission 308 nm	Chiral AGP (150 × 4.0 mm, 5 µm), CSP: α_1_-acid glycoprotein	human plasma LLE	verapamil N-acetyl-norverapamil	[12]
mobile phase: 0.01 M phosphate buffer (pH 7.0):acetonitrile (90:10, *v*/*v*), fluorescence detection: excitation 276 nm, emission 310 nm	Chiral–AGP (100 × 4 mm, 5 µm), CSP:α_1_-acid glycoprotein	human serum online SPE	verapamil, norverapamil, D617; D620; PR24; PR23; PR22; PR25 metabolites	[14]
mobile phase: 25 mm phosphate buffer) (pH 6):isopropanol (85:15, *v*/*v*), flow rate = 0.9 mL/min, coulometric detection	Chiral CBH (100 × 4.0 mm, 5 µm), CSP: cellobiohydrolase	microsomal incubation mixtures LLE	salmeterol α-hydroxysalmeterol	[15]
mobile phase: *n*-hexane:isopropanol (98:2, *v*/*v*), flow rate = 0.8 mL/min, 25 °C, UV detection 215 nm	Chiralcel OD column (250 × 4.6 mm, 5 µm) CSP: cellulose tris(3,5-dimethylcarbammate)	human plasma LLE	ketamine, norketamine bromoketamine (I.S.)	[16]
mobile phase: *n*-hexane:ethanol:isopropanol (18:2:1, *v*/*v*/*v*) + 0.1% of glacial acetic acid, flow rate = 1.0 mL/min, 25 °C, UV detection 215 nm	Chiralcel OD column (250 × 4.6 mm) CSP: cellulose tris(3,5-dimethylphenylcarbamate)	human urine LLE	10-hydroxycarbazepine carbamazepine-10,11-transdihydrodiol oxcarbazepine oxime (achiral I.S)	[17]
mobile phase: gradient elution: solvent A (acetonitrile:0.01 M citrate buffer (pH 2.7):0.05 M perchlorate buffer (pH 2.0) (50:25:25 *v*/*v*/*v*), solvent B (acetonitrile), flow rate = 1 mL/min, 60 °C, fluorescence detection: excitation 325 nm, emission 430 nm	Chiralcel OD-R (250 × 4.6 mm, 5 μm) CSP: cellulose tris (3,5-dimethylphenylcarbamate)	plasma LLE	fenfluramine, norfenfluramine, phentermine (achiral)	[18]
mobile phase: acetonitrile:water, (75:25, *v*/*v*), flow rate = 0.8 mL/min, MS detection	Chirobiotic T (250 × 4.6 mm, 5 µm), CSP: teicoplanin	bulk	15 different amino acids	[19]
mobile phase: 40 mM potassium dihydrogen phosphate buffer (pH 5.5):acetonitrile (91:9, *v*/*v*), flow rate = 0.8 mL/min, 20 °C, fluorescence detection: excitation 286 nm, emission 320 nm	ChiralDex (250 × 4 mm, 5 µm), CSP: β-cyclodextrin	human plasma SPE	N-ethyl-3,4-methylenedioxyamphetamine 3,4-methylenedioxyamphetamine	[20]
mobile phase: methanol:water (75:25, *v*/*v*), flow rate = 0.5 mL/min, UV detection 285 nm.	Chiralcel OD-R (250 × 4.6 mm, 5 μm) CSP: cellulose tris(3,5-dimethylphenylcarbamate)	human liver microsomes LLE	lansoprazole, 5-hydroxy-lansoprazole, lansoprazole-sulfone (achiral)	[21]
mobile phase: isopropanol, flow rate = 0.5 mL/min, UV detection 214 nm	homemade cellulose tris (3,5-dichlorophenylcarbamate) CSP (250 × 4.6 mm)	bulk	*cis*-diltiazem, desacetyl-*cis*-diltiazem	[22]
mobile phase: 20 mM phosphate buffer (pH 6.44) containing 50 mM EDTA:isopropanol (93:7, *v*/*v*), flow rate = 0.7 mL/min, 17.5 °C, fluorescence detection: excitation 286 nm, emission 322 nm	Chiral CBH (150 × 4 mm, 5 μm) CSP: cellobiohydrolase	human plasma, human urine SPE	N-ethyl-3,4-methylenedioxyamphetamine, N-ethyl-4-hydroxy-3-methoxyamphetamine, 3,4-methylenedioxyamphetamine	[23]
mobile phase: 100 mm potassium hexafluorophosphate (pH 3.0):acetonitrile (65:35, *v*/*v*), flow rate = 0.5 mL/min, 37 °C, UV detection 227 nm	Chiralcel OD-R column (250 × 4.6 mm, 10 μm) CSP: cellulose tris(3,5-dimethylphenylcarbamate)	plasma LLE	fluoxetine, norfluoxetine, fluvoxamine (achiral I.S.)	[24]
mobile phase: 0.5 M sodium perchlorate:acetonitrile:methanol (6:3:1, *v*/*v*/*v*), flow rate = 0.5 mL/min, ambient temperature, UV detection 285 nm	Chiral CD-Ph (250 × 4.6 mm, 5 μm), CSP: phenylcarbamated β-CD	human plasma SPE	lansoprazole, 5-hydroxy-lansoprazole, lansoprazole sulfone (achiral), *S*-omeprazole (I.S.)	[25]
mobile phase: 0.5 M sodium perchlo-rate:acetonitrile (6:4, *v*/*v*), flow rate = 0.5 mL/min, ambient temperature, UV detection 285 nm	Chiral CD-Ph (250 × 4.6 mm, 5 μm), CSP: phenylcarbamated β-CD-based	human plasma SPE	rabeprazole, rabeprazole-thioether (achiral), rabeprazole sulfone (achiral), *S*-omeprazole (I.S.)	[26]
mobile phase: hexane:isopronaol:methanol:diethylamine (75:10:15:0.1, *v*/*v*/*v*/*v*), flow rate =1.0 mL/min, 25 °C, UV detection 225 nm	Chiralpak AD-H (250 mm × 4.6 mm, 5 µm), CSP: amylose tris-(3,5-dimethylphenylcarbamate)	tablets	zolmitriptan, impurity-1	[27]
mobile phase: hexane:isopropanol:diethylamine: 0,1% trifluoroacetic acid (85:15:0.15:0.2, *v*/*v*/*v*/*v*), flow rate = 1.0 mL/min, ambient temperature, UV detection 273 nm	Chiralpak OD-H column (250 × 4.6 mm, 5 μm) CSP: cellulose tris(3,5-dimethylphenylcarbamate)	bulk	atomoxetine, meta- and para-isomers of atomoxetine and desmethyl-atomoxetine	[28]
mobile phase: water:methanol (88:12, *v*/*v*), flow rate = 0.7 mL/min, 30 °C, UV detection 225 nm	Lichro-CART 250-4 ChiraDex (250 × 4 mm, 5 µm) CSP: β-cyclodextrin	mouse plasma and brain, liver, and kidney tissue homogenates, SPE	eslicarbazepine acetate, licarbazepine, oxcarbazepine (achiral)	[30]
mobile phase: 0.4% trifluoroacetic acid:acetonitrile (80:20, *v*/*v*), flow rate = 0.8 mL/min, 30 °C, UV detection 225 nm	Cyclobond I 2000 DM (250 × 4.6 mm, 5 µm) CSP: dimethyl β-cyclodextrin	bulk, capsules, tablets	sertraline and its 9 process-related impurities	[31]
mobile phase: methyl-tert-butyl ether:ethyl acetate:ethanol:diethylamine (60:40:5:0.1, *v*/*v*/*v*/*v*), flow rate: 1 mL/min (analytical) and 4 mL/min (semipreparative), 25 °C, UV detection 310 and 285 nm, circular dichroism detection 285 nm	Chiralpak IA (250 × 4.6 mm, analytical), (250 × 10 mm, semipreparative), CSP: amylose tris (3,5-dimethyl-phenylcarbamate)	bulk	lansoprazole, impurities A, B–E (achiral)	[32]
mobile phase: methyl tert-butylether:ethyl acetate:ethanol:diethylamine (60:40:5:0.1, *v*/*v*/*v*/*v*), flow rate = 1 mL/min (analytical), 4 mL/min (semipreparative), 25 °C, UV and circular dichroism detection 299 nm	Chiralpak IA (250 × 4.6 mm, analytical), (250 × 10 mm, semipreparative) CSP: amylose tris (3,5-dimethyl-phenylcarbamate)	bulk, tablets	esomeprazole, impurities B, F, H, and impurities A, C, D, F, G, and I (achiral)	[34]
mobile phase: phosphate buffer (pH–5.0):methanol (45:55, *v*/*v*), flow rate = 0.4 mL/min, UV detection 254 nm	Chiral CD-ph (150 mm × 4.6 mm, 5 μm) CSP: phenylcarbamated β-CD-based	human plasma LLE	omeprazole, 5-hydroxy-omeprazole, omeprazole sulfone (achiral)	[36]
mobile phase: ethanol:water (100:60, *v*/*v*), acetonitrile:water (100:100, *v*/*v*) (fenbendazole), flow rate = 1.0 mL/min, 25 °C, UV detection 254 nm	Chiralpak IA-3 (100 mm × 4.6 m, 3 μm) CSP: amylose-tris(3,5-dimethylphenylcarbamate)	bulk	albendazole (achiral), albendazole-sulfone (achiral), albendazole-sulfoxide, fenbendazole (achiral), fenbendazole-sulfone (achiral), and fenbendazole-sulfoxide	[37]
mobile phase: phosphate buffer (pH–8.0):acetonitrile-gradient elution, flow rate = 1.0 mL/min, 35 °C. UV detection 282 nm	Chiralpak IC column (250 mm × 4.6 mm, 5 μm) CSP: cellulose tris-(3,5-dichlorophenylcarbamate)	bulk	rabeprazole, impurities A, B, C (achiral), and impurities D and E	[38]
mobile phase: acetonitrile:methanol:acetic acid:diethylamine (95:5:0.2:0.07, *v*/*v*/*v*/*v*), flow rate = 1.0 mL/min, 40 °C, UV detection 240 nm.	Sepapak-2 (250 mm × 4.6 mm, 5 µm) CSP: cellulose tris(3-chloro-4-methylphenylcarbamate)	microsomal incubation mixtures SPE	eslicarbazepine acetate licarbazepine, oxcarbazepine (achiral)	[39]
mobile phase: analytical: ethanol:water (50:50, *v*/*v*), flow rate = 1 m/min, 25 °C, UV detection 280 nm mobile phase: semipreparative: mobile phase: *n*-hexane:ethanol:diethylamine (40:60:0.1, *v*/*v*/*v*) (Impurity A), *n*-hexane:ethanol:diethylamine (60:40:0.1 *v*/*v*/*v*) (lansoprazole), flow rate = 4.5 mL/min, 35 °C, UV detection 310 nm	Chiralpak IC-3 (100 × 4.6 mm, analytical) CSP: cellulose tris-(3,5-dichlorophenylcarbamate)	bulk	lansoprazole, impurities A, B–E (achiral)	[41]
mobile phase: acetonitrile:water (50:50, *v*/*v*), flow rate = 1 mL/min, 40 °C, UV detection 280 nm and 300 nm, circular dichroism detection 280 nm	Chiralpak ID-3 (100 × 4.6 mm, 3 μm) CSP: amylose tris-(3-chlorophenylcarbamate)	bulk	esomeprazole, impurities B, E, F (*R*-omeprazole), impurities A, C, D (achiral)	[42]
mobile phase: analytical: methanol:water (100:15, *v*/*v*), semipreparative: *n*-hexane:ethanol (70:30, *v*/*v*); flow rate = 4.5 mL/min, 25 °C; UV detection 280 nm	Chiralcel OJ-RH (150 mm × 4.6 mm, 5 μm, analytical), (250 mm × 10 mm, 5 μm, semipreparative), CSP: cellulose tris(4-methylbenzoate)	bulk, tablets	clopidogrel, impurities B, C	[43]
mobile phase: 20 mM ammonium acetate:methanol (10:90, *v*/*v*), flow rate = 0.4 mL/min, 25 °C, MS (MRM) detection	Chirobiotic T column (250 mm × 4.6 mm, 5 μm) CSP: teicoplanin	rat plasma LLE	bambuterol, terbutaline	[44]
mobile phase: 0.1% diethylamine in methanol, flow rate = 1.0 mL/min, ambient temperature, UV detection at 254 nm	Lux Cellulose-3 (250 mm × 4.6 mm, 3 µm) CSP: cellulose tris(4-methylbenzoate)	bulk, tablets, injection solution	levomepromazine, dextromepromazine, levomepromazine sulfoxide, 2-methoxyphenothiazine (achiral)	[45]
mobile phase: ethanol:water-gradient elution, flow rate = 1.0 mL/min, UV detection 270 nm	Lux Cellulose-1 (50 × 4.6 mm, 5 μm) CSP: cellulose tris(3,5-dimethylphenylcarbamate)	bulk, pharmaceutical syrup,	guaifenesin, ambroxol	[46]
mobile phase: 10 mM acetate buffer (pH–5.0):acetonitrile (65:35, *v*/*v*), flow rate = 0.4 mL/min, 25 °C, MS (MRM) detection	Chiralpak AD-RH (150 × 4.6 mm, 5 μm) CSP: amylose tris(3,5-dimethylphenylcarmabate)	surface water sample SPE	flurbiprofen, ibuprofen, naproxen	[48]
mobile phase: acetonitrile:water:diethylamine (75:25:0.1, *v*/*v*/*v*), flow rate = 1.0 mL/min, 30 °C	Chiralpak IG-3 (250 mm × 4.6 mm, 3 μm) CSP: amylose tris(3-chloro-5-methylphenylcarbamate)	bulk, tablets	sertraline, impurities A, B, C, E, F, and G	[49]
mobile phase: *n*-hexane:isopropanol: trifluoroacetic acid (80;20:0.1, *v*/*v*/*v*), flow rate = 1 mL/min, 5 °C, UV detection 330 nm	Chiralpak AS-H (250 mm × 4.6 mm, 5 μm) CSP: amylose tris[(*S*)-α-methylbenzylcarbamate]	bulk	α-lipoic acid, α-dihydrolipoic acid	[50]
mobile phase: ethanol:diethylamine:water-gradient elution and flow-programming, 40 °C, UV detection 210 nm and 224 nm	Lux Cellulose-3 (150 × 4.6 mm, 5 μm) CSP: cellulose tris(4-methylbenzoate)	bulk, tablets	dapoxetine, dapoxetine impurities, including R-dapoxetine, (3 S)-3-(dimethylamino-3-phenyl-1-propanol), S-3-amino-3-phenyl-1-propanol, 1-naphtol (achiral), 4-phenyl-2H,3H,4H-naphtho[1,2- b ]pyran (achiral), 1-(2 E)-Cinnamyloxynaphthalene (achiral)	[51]
mobile phase: acetonitrile:methanol (98:2, *v*/*v*) containing 0.06% DEA, flow rate = 0.45 mL/min, 12 °C, UV detection 286 nm	Lux Cellulose-2 (4.6 × 150 mm, 5 µm) CSP: cellulose tris(3-chloro-4-methylphenylcarbamate)	bulk, tablets	ivabradine, dehydro-S-ivabradine, N-demethyl-*S*-ivabradine, ((*S*)-3,4-dimethoxy-bicyclo[4.2.0]octa-1,3,5-triene-7-yl-methyl)-methyl-amine), 1-(7,8-dimethoxy-1,3,4,5-tetrahydro-2H-3-benzazepine-2-on-3-yl)-3-chloro-propane) (achiral)	[52]
mobile phase: analytical: ethanol:water:diethylamine (80:20:0.1, *v*/*v*/*v*), mobile phase semipreparative: ethanol, flow rate = 0.5 mL/min, 35 °C, UV detection 295 nm, 263 nm (impurity I), and 243 nm (impurity G)	Chiralpak IA-3 (250 mm × 4.6 mm, 3 μm—analytical) (250 mm × 10 mm—semipreparative) CSP: amylose-tris(3,5-dimethylphenylcarbamate)	bulk	paroxetine, impurities A, C, D, E, I, impurity G, sesamol (achiral)	[53]
mobile phase: water:acetonitrile:diethylamine (55:45:0.01, *v*/*v*/*v*), flow rate = 0.8 mL/min, 25 °C, UV detection 230 nm	Lux Cellulose-1 (150 × 4.6 mm, 5 µm) CSP: cellulose tris(3,5-dimethylphenylcarbamate)	bulk, tablets	escitalopram, *R*-ciralopram, impurities C, D, E	[55]
NP method: mobile phase: *n*-hexane:ethanol:diethylamine 90:10:0.1 (*v*/*v*/*v*), flow rate = 1.0 mL/min, 30 °C; UV detection 240 nm RP method: mobile phase: ethanol:water:diethylamine (70:30:0.1, *v*/*v*/*v*), flow rate = 0.4 mL/min, 30 °C, UV detection 240 nm	Chiralcel OJ-H (250 mm × 4.6 mm, 5 μm) CSP: cellulose tris(4-methylbenzoate)	bulk	escitalopram, impurities A, B, C, H, and K	[56]
mobile phase: acetonitrile:trifluoracetic acid (0.05%)-gradient elution, flow rate = 1.0 mL/min, 40 °C; UV detection 240 nm	Lux Cellulose-2 (250 mm × 4.6 mm, 3 μm) CSP: cellulose tris(3-chloro-4-methylphenylcarbamate)	bulk, tablets	rosuvastatin, impurities A, B, C, D, G, FP-A, FP-B	[57]
mobile phase: methanol:water:diethylamine (80:20:0.2, *v*/*v*/*v*), flow rate = 0.45 mL/min, 45 °C, UV detection 215 nm	Lux Cellulose-2 (250 ×4.6 mm, 5 μm) CSP: cellulose tris(3-chloro-4-methylphenylcarbamate)	bulk, tablets	vildagliptin, *R*-vildagliptin, dehydroxy-vildagliptin, vildagliptin-dimer, vildalgliptin-amide, vildalgliptin-diketopiperazine (achiral)	[58]

## Data Availability

Data are contained within the article.

## Abbreviations

CD	cyclodextrin
CE	capillary electrophoresis
CS	chiral selector
CSP	chiral stationary phase
EMA	European Medical Agency
ESI	electrospray ionization
Eur Ph	European Pharmacopoeia
FDA	Food and Drug Administration
HILIC	hydrophilic interaction chromatography
HPLC	high-performance liquid chromatography
I.S	internal standard
LLE	liquid–liquid extraction
LOD	limit of detection
LOQ	limit of quantification
MRM	multiple reaction monitoring
MS	mass spectrometry
NP	normal phase
PI	polar ionic
PO	polar organic
PPI	proton pump inhibitor
RP	reverse phase
SPE	solid phase extraction
SSRI	selective serotonin reuptake inhibitor
USP	United States Pharmacopoeia
